# Does acute pulmonary vein stenosis secondary to edema on immediate post ablation procedure CMR increase risk for development of chronic pulmonary vein stenosis

**DOI:** 10.1186/1532-429X-14-S1-P211

**Published:** 2012-02-01

**Authors:** Dan Sommers, Eugene Kholmovski

**Affiliations:** 1 Radiology, University of Utah, Salt Lake City, UT, USA

## Summary

Our hypothesis is that patients who demonstrate acute pulmonary vein narrowing or stenosis secondary to edema following radiofrequency ablation (RFA) procedure for atrial fibrillation as demonstrated on immediate post procedural cardiac magnetic resonance (CMR) examination are at an increased risk for developing chronic pulmonary vein stenosis at 3 month followup CMR.

## Background

From January, 2010 to September, 2011, 224 patients who underwent pulmonary vein isolation and debulking of the septal and posterior walls of the left atrium as part of RFA procedure for atrial fibrillation were imaged on a 3 Tesla MR scanner (Verio, Siemens Healthcare). Time interval between the conclusion of ablation procedure and placing the patient in the scanner was typically less than one hour. The MR protocol included double inversion recovery T2-weighted TSE, contrast enhanced MR angiography (Multihance, 0.1 mmol/kg) and 3D late gadolinium enhancement (LGE) scans.

## Methods

Retrospective review of records was performed to determine those patients in whom acute pulmonary vein narrowing or stenosis was identified on their immediate post RFA procedure CMR examination. Prospective followup of these identified patients was then performed to determine those patients who went on to develop chronic pulmonary vein stenosis at 3 month followup CMR imaging.

## Results

Data collection is ongoing. Currently 30 patients have been identified as having findings of acute pulmonary vein narrowing or stenosis secondary to edema on their immediate post procedural CMR. Sixteen of these patients have had three month followup CMR examination. Twelve patients have pending 3 month followup imaging scheduled within the next several months. Two patients have been lost to followup to date. Of the 16 patients found to have acute pulmonary vein stenosis secondary to post procedural edema per CMR, and who have had a 3 month followup to date, 13 of the 16 patients (81%) demonstrate chronic pulmonary vein narrowing or stenosis at 3 month followup examination. Additional patients and followup data will be included in the final results. Results will include extent of immediate post procedural and chronic pulmonary vein narrowing and stenosis.

## Conclusions

Findings of acute pulmonary vein narrowing or stenosis on immediate post radiofrequency ablation procedures for history of atrial fibrillation have an increased risk of developing chronic pulmonary vein stenosis at 3 month followup CMR examination. It has previously been demonstrated that no patient with a normal examination at 3 month post RFA procedure demonstrated significant stenosis at 6 to 12 months.

## Funding

No grants or additional funding was obtained.

**Figure 1:**
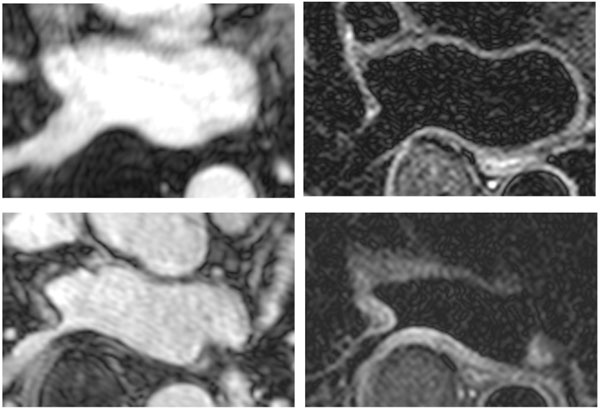
Upper Row: Pre-ablation MRA and dark-blood TSE of Right Inferior Pulmonary Vein (RIPV); Lower Row: Immediate Post-ablation MRA and dark-blood TSE of RIPV.

